# Danish translation, cultural adaption and initial psychometric evaluation of the patient feedback form

**DOI:** 10.1186/s12955-018-0900-4

**Published:** 2018-04-27

**Authors:** Lærke K. Tolstrup, Helle Pappot, Graziella Zangger, Lars Bastholt, Ann-Dorthe Zwisler, Karin B. Dieperink

**Affiliations:** 1 Department of Oncology, Odense University Hospital, Odense, Denmark; 2 Department of Oncology, Copenhagen University Hospital, Copenhagen, Denmark; 3 The Danish Knowledge Centre for Rehabilitation and Palliative Care, University of Southern Denmark, and Odense University Hospital, Odense, Denmark; 4 University of Southern Denmark, Odense, Denmark

**Keywords:** Questionnaire, Translation, Validation, Psychometric testing, Patients reported experience measures, PREM, Patients reported outcome measures, PROM

## Abstract

**Aim:**

No suitable Danish questionnaire exists to evaluate patient satisfaction with various patient reported outcome measures. Thus, the aim of this research project was to conduct a study on the translation and cultural adaption of an American patient reported experience measures questionnaire, “Patient Feedback Form”, among Danish patients, and to examine selected psychometric properties within reliability.

**Material and methods:**

In the first phase of the study, the Patient Feedback Form was forward and backward translated following the methodology of existing guidelines. Subsequently, cognitive interviewing was performed with seven cancer patients and seven healthy persons (19–86 years old/6 men and 8 women) to ensure that questions were easy to understand and made sense to Danish interviewees.

In the second phase, phone interviews were carried out with 95 prostate cancer patients after they had responded to the same Patient Feedback Form. Missing data was imputed using the Expectation-Maximization technique. To examine the structure of the questionnaire, an exploratory factor analysis was conducted. Cronbach’s alpha was calculated to investigate internal consistency.

**Results:**

There were only minor disagreements in the translation process, and the reconciliation went smoothly (phase 1). With regard to one item, however, it was difficult to reach a consensus. Through the qualitative validation process, the right solution was found. The results from the psychometric testing (phase 2) showed that four factors had an Eigen value > 1, but only one factor was extracted as the Scree plot had a clear “elbow”, showing a one factor structure that explained 46.1% of the variance. The internal consistency was high as Cronbach’s alpha was 0.89.

**Conclusion:**

The translated, culturally adapted, and validated version of the Patient Feedback Form seems to be suitable for measuring satisfaction with patient reported outcome measures in a Danish setting. While the results should be treated with caution due to the small sample size, psychometric testing indicates that the questionnaire is a valid instrument. However, additional psychometric testing such as hypotheses testing, responsiveness, and test-retest on a larger and more diverse sample size is required to further verify the validity of the instrument.

## Introduction

Several questionnaires to measure patient reported outcome measures (PROMs) exist and have become an increasingly popular source for collecting information on patient conditions, e.g. physical symptoms, toxicities, or psychosocial problems [1]. Some instruments are generic, dealing with issues such as quality of life(QoL), anxiety, depression, and pain, while others are disease-specific [2]. In the past, PROMS have mainly been used in clinical trials to determine safety, efficacy and cost effectiveness of, for example, a new drug [3, 4]. Thus, the data collected in research settings has generally not been available to clinicians [5]. In many cases, the questionnaires have been independent tools that have helped the health care system gain knowledge of, for example, patients´ symptoms and QoL on a general level. Fortunately, PROMs have also moved into the world of routine care – probably eased by electronic data collection – where they are integrated into the patient trajectory with the purpose of influencing treatment and care. In some circumstances, the results are provided to clinicians to improve patient care and focus on patient concerns [4, 5]. Little is known, however, about the value of this integration from a patient perspective or how patients experience filling out the questionnaires. Thus, it is important to explore if the patients found the questionnaire easy to complete, if it improved patient-clinician communication and/or enhanced quality of care. These are relevant issues to examine at a time when focus on patient reported experiences and attention to patient involvement and satisfaction have increased and are mandatory in many health care settings. More research is needed on the effects of PROM interventions in different settings [6–8] and to establish what realistic benefits can be gained from using PROMs in routine care [9]. Using a Patient Reported Experience Measures questionnaire (PREM-questionnaire) to evaluate if a given PROM is worthwhile [5], and/or to identify which PROM(s) to use [4, 10], may be one method to select feasible and patient-acceptable PROMs.

**Fig. 1:**
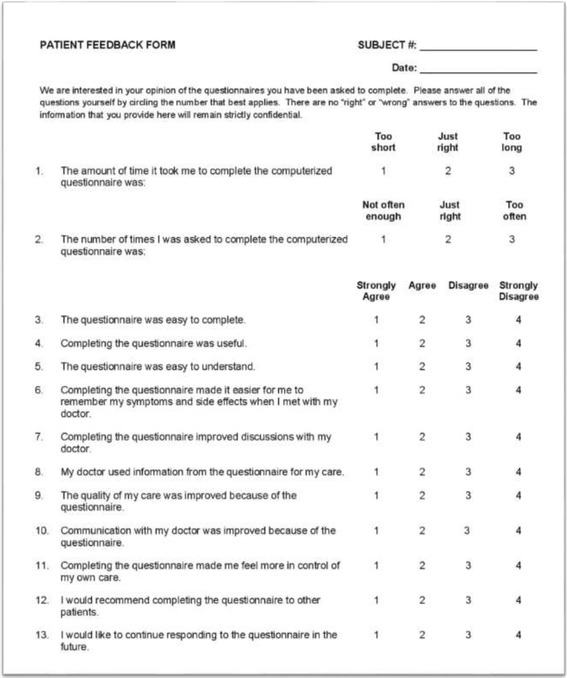
Patient Feedback Form: Developed by Ethan Basch: Adapted by Claire Snyder

Since no suitable PREM-questionnaire was available in Danish, an American questionnaire entitled “Patient Feedback Form” was chosen [4, 5, 11]. The Patient Feedback Form was selected because it evaluates the usefulness and value of a given PROM from the patient perspective. Thus, the Patient Feedback Form is relevant in situations where the health care system wishes to examine patient satisfaction with PROMs that are integrated into clinical practice. Furthermore, the Patient Feedback Form is short and, due to its generic nature, we expected it to be adaptable to a Danish setting and useful in many different areas within the health care system. To our knowledge, the form has not been translated into other languages. The questionnaire consists of 13 items (Fig. 1). Respondents evaluate their level of agreement/disagreement on a scale with four options to eliminate the neutral response [12]. Two questions have a 3-point option. The Patient Feedback Form has not undergone any traditional psychometric testing in the original language.

Firstly, the aim of this study was to translate and culturally adapt the questionnaire into Danish following existing guidelines [13, 14] and, secondly, to carry out initial psychometric evaluation.

## Materials and methods

### Phase 1 – The translation and cultural adaption process

#### Preparation and approvals

An expert group was formed to oversee the translation process. The group consisted of a senior oncologist [15] and a senior nurse who both had experience with translations and cross-cultural adaptions and the project manager.

Permission to translate the Patient Feedback Form was granted from the developer, Ethan Basch [11], and Claire Snyder [4, 5], who had adapted the questionnaire. According to Danish law, approval from the ethics committee was not required, but the study was registered with the Danish Data Protection Agency.

#### Forward translation and reconciliation

The Patient Feedback Form was translated into Danish by two independent, experienced translators, who had Danish as their mother tongue, were fluent in English [13], and had been residents in an English speaking country for more than two years. They did not have a medical background, which was acceptable because the questionnaire does not contain medical language, health care terminology, or require any particular knowledge. Focus was kept on the natural, spoken language with its cultural nuances addressing a common audience [16].

Comparisons were made between the independent translations regarding ambiguity and discrepancies of words, sentences, or meaning for each item in the questionnaire in order to create a consensus version.

#### Backward translation and review

The Danish consensus version was back-translated by two independent bilingual translators blind to the original. The translators had English as their mother tongue but had resided in Denmark for several years. As with the forward translations, the translators were asked to take a conceptual approach due to the subjective nature of the construct (patient experience and satisfaction) [13].

The two translations were then compared to the original to ensure that the translated versions reflected the same item content.

#### Pre-testing/pilot testing

Cognitive interviewing was performed with 7 cancer patients receiving immunotherapy for malignant melanoma and 7 healthy persons (19–86 years/6 men and 8 women). The respondents were selected to ensure an equal distribution across age and gender**.** A combination of the “think aloud” method and “probing” was applied [14] to ensure that the items were easy to understand and made sense to a Danish population. Proofreading was performed and a report sent to the developer and adaptor [13].

### Phase 2 – Psychometric testing

There are no general criteria for calculating sample size when assessing internal consistency and factor analysis. The Cosmin guideline, however, contains standards for evaluating the methodological quality of studies on measurement properties [17]. According to the Cosmin checklist, a sample size of minimum 100 respondents or seven respondents times the number of items is recommended [17]. A convenience sample of 102 men with prostate cancer in post-treatment control (54–73 years old) were chosen as respondents because they all had filled out the same PROM-questionnaire concerning satisfaction with treatment and care, and were available as respondents. In total, 95 (93%) accepted the invitation to respond. Not all of the patients had experienced any problems during their post-treatment control and as a consequence, they had not been in contact with a health care professional. Accordingly, they were not able to answer the items in the Patient Feedback Form which deal with this interaction.

In the original version, the Patient Feedback Form was used in connection with cancer patients [4, 5, 11], which explains why we selected this group of patients for psychometric evaluation. The respondents were interviewed over the phone. Phone interviews were chosen to motivate respondents to answer and to facilitate conducting the survey within a short period of time. An expert on questionnaire technique was consulted to make sure that the questionnaire was adapted to the chosen survey format. Consequently, I, me and my were exchanged with you and yours during the interviews. Moreover, a guideline was designed [18] to make the interaction as smooth as possible. The interviews were carried out by the same interviewer to ensure uniformity.

The structure (i.e. the number of factors) of the Patient Feedback Form was unknown, and it was not possible to make a confirmatory factor analysis because no psychometric testing of the original version had been carried out. Thus, the psychometric evaluation comprised of an exploratory factor analysis (EFA) if the Kaiser-Meyer-Olkin (KMO) measure of sampling adequacy was > 0.6 and if the Bartlett’s test of sphericity was significant (*p* < 0.05) [19]. The number of latent factors were decided by evaluating the scree plot and the number of factors with Eigenvalues > 1. The EFA method and rotation of the factors were chosen depending on the number of factors in the initial EFA. If one factor (as expected) was extracted, the maximum likelihood extraction method without rotation was applied [19]. Further, to assess internal consistency, Cronbach’s Alpha (α) was evaluated. The level of α was considered: fair = 0.70–75; moderate = > 0.75–0.80; good = > 0.80–0.85; excellent > 0.85–0.90 [20]. Missing data was assessed by Little’s Missing Completely at Random (MCAR) test [21]. If participants had > 3 missing items (aside from the five items concerning interaction with healthcare professionals), they were excluded from the analysis. In the case of missing data and a non-significant (*p* > 0.05) MCAR test, the Expectation-Maximization (EM) technique was used to impute data [21]. A significant level of 0.05 was chosen and all analyses were executed using SPSS version 23.

## Results

### Phase 1 – The translation and cultural adaption process

Overall, consensus was easy to achieve and neither the translators nor the experts felt that they had to compromise. As for the forward translation, minor discrepancies such as the use of synonyms – digital vs. electronic – and different word order were detected. One of the translators, for example, suggested, “Completing the questionnaire improved discussions with my doctor” whereas the other suggested, “Discussions with my doctor were improved because I had completed the questionnaire.” Also, the back-translated versions were close to the original. In the original version, the word “completed” was used for filling out the questionnaire whereas the two backward translators had chosen “answer” and “respond to”. However, it was not possible to reach a consensus on whether or not the English loanword “feedback” should be translated into Danish. The expert group decided to leave it up to the pilot testing, resulting in the word being translated into a Danish word. Also, the respondents found two items (Fig. 1, items 7 and 10) to be almost identical. However, in order to be true to the original, nothing was changed. With regard to item 11, the semantics was changed somewhat. The phrase “Control of”´ did not sit well with the Danish patients, who did not feel it was in their power to be in control – nor did they want to be. “That is the doctor’s job,” as one respondent put it. Instead of control, the Danish respondents suggested the word “involved”, which they found more appropriate. The Danish version was adapted accordingly. Furthermore, the word doctor was changed to healthcare professional to broaden the scope of the questionnaire. All changes were approved by the developer.

### Phase 2 - psychometric testing

Of the 95 respondents, 56 respondents (58.9%) were not able to answer all 13 items since they had not been in contact with a healthcare professional; five of the items (Fig. 1, items 6–10) deal with this interaction. Moreover, two respondents had > 3 items missing (when the items about interaction with a healthcare professional where not included) and, therefore, they were excluded (Table 1). The MCAR test showed that data was missing completely at random (*p* = 0.307). The missing data was replaced by the EM method. The EFA was conducted as the KMO was 0.731 and Bartlett’s test significant (*p* < 0.001). Four factors had an Eigen value > 1, but only one factor was extracted as the Scree plot had a clear “elbow”, showing one factor explaining 46.1% of the variance. Three items had a factor load < 0.4, (Table 2). The internal consistency was high as Cronbach’s α was 0.89. The inter-item correlations ranged widely between − 0.001-0.773, with items 2 and 5 showing the lowest correlation and items 10 and 11 the highest (Table 3).

**Table 1:** Item statistics and percentage of missings per item of the Patient Feedback Form

Item	N	Mean	SD	Missing	
				Count	Percent
1: Time it took completing	93	2.12	0.357	2	2.1
2: Number of time completing	92	1.97	0.346	3	3.2
3: Easy to complete	94	1.74	0.671	1	1.1
4: Completion was useful	95	1.69	0.745	0	0.0
5: Easy to understand	95	1.67	0.643	0	0.0
6: Easier to recall symptoms and side effects	38	2.00	0.805	57	60.0
7: Improved discussions with clinician	37	1.95	0.780	58	61.1
8: Clinician used information for care	33	1.85	0.870	62	65.3
9: Care quality improved	31	2.26	0.815	64	67.4
10: Communication with clinician improved	35	2.09	0.919	60	63.2
11: Made me more in control of care	94	1.79	0.760	1	1.1
12: Recommend to other patients	93	1.30	0.484	2	2.1
13: Want to continue using	90	1.28	0.450	5	5.3

N, numbers; SD, Standard deviation

**Table 2:** Factor matrix and item statistics with no missings from the Patient Feedback Form

Item	Factor	Mean	SD
1: Time it took completing	0.333	2.12	0.358
2: Number of time completing	0.132	1.97	0.345
3: Easy to complete	0.307	1.75	0.670
4: Completion was useful	0.568	1.70	0.749
5: Easy to understand	0.594	1.67	0.631
6: Easier to recall symptoms and side effects	0.573	1.95	0.669
7: Improved discussions with clinician	0.922	1.86	0.598
8: Clinician used information for care	0.836	1.79	0.627
9: Care quality improved	0.807	2.12	0.576
10: Communication with clinician improved	0.746	2.01	0.750
11: Made me more in control of care	0.858	1.78	0.764
12: Recommend to other patients	0.656	1.32	0.511
13: Want to continue using	0.568	1.29	0.463

SD, Standard deviation

**Table 3:** Inter-item correlation matrix of the Danish version of the Patient Feedback Form

Item	1	2	3	4	5	6	7	8	9	10	11	12	13
1: Time it took completing	1.000	0.302	0.268	0.439	0.180	0.166	0.302	0.095	0.264	0.137	0.295	0.454	0.392
2: Number of time completing		1.000	0.169	0.141	***−0.001***	0.223	0.058	0.044	0.150	0.116	0.054	0.246	0.313
3: Easy to complete			1.000	0.413	0.466	0.166	0.229	0.237	0.329	0.009	0.210	0.289	0.319
4: Completion was useful				1.000	0.406	−0.024	0.479	0.462	0.704	0.103	0.475	0.506	0.580
5: Easy to understand					1.000	0.595	0.616	0.415	0.398	0.423	0.413	0.396	0.308
6: Easier to recall symptoms and side effects						1.000	0.587	0.476	0.250	0.637	0.448	0.441	0.215
7: Improved discussions with clinician							1.000	0.768	0.747	0.728	0.770	0.573	0.513
8: Clinician used information for care								1.000	0.772	0.651	0.742	0.509	0.334
9: Care quality improved									1.000	0.473	0.695	0.482	0.454
10: Communication with clinician improved										1.000	***0.773***	0.413	0.253
11: Made me more in control of care											1.000	0.536	0.510
12: Recommend to other patients												1.000	0.750
13: Want to continue using													1.000

Bold and italic for highest and lowest correlation

## Discussion

Overall, the translated version was equivalent to the original version with only minor changes. However, one item had to be changed due to cultural differences. The results from the psychometric testing supported a one factor-structure and showed a high internal consistency (0.89) in the final Danish version.

In the forward translation, both translators had chosen not to translate the English word “feedback” in the title. The word is a loanword in Danish and the translators believed that the word was so integrated into the Danish language that everyone would understand the meaning. The respondents disagreed on whether or not it was appropriate in the Danish version since there was a risk that older patients in particular would not understand it. Consequently, we decided to choose the Danish word “tilbagemelding” – the best possible translation of feedback – which was also suggested by some of the respondents. Concerning items 7 and 10, which were found to be similar, it might be argued that future respondents may find it annoying that two items are almost identical. However, there are some nuances. The word “discussions” may, for example, be more of an active exchange of opinions between patient and physician whereas “communication” may also be one-sided with the physician setting the agenda. Moreover, the importance of staying true to the original was prioritized. An inter-item correlation of 0.728 supports the argument that, despite the similarity, the items are not redundant. As for the phrase “control of”, which the Danish respondents disapproved of, we decided that cultural adaption was more important than sticking to the original phrase. Due to cultural differences, it may be more natural for American patients to feel in control of treatment and care [22], whereas the cognitive interviewing suggests that Danish patients prefer to be actively engaged in the process, which is also supported by the patient organization Danish Patients [23]. Accordingly, the wording was changed. Similar cultural adaptions are found in other questionnaire translations [15].

Far from all patient satisfaction questionnaires have undergone psychometric testing [2], which is also the case for the original version of this questionnaire. However, initial psychometric testing of the translated version shows satisfactory results. The EFA reveals a one factor latent structure. As less than half of the variance (46.1%) is explained by one factor, the presence of two factors could be discussed. One factor focused on the feasibility of completing the PROM and the other focused on the clinical utility of the questionnaire in the process of health care. Internal consistency is defined as the degree of relation between items [12], and the high Cronbach’s α (0.89) supports the results of a one factor structure. However, the possibility of an artificially increased Cronbach’s α is present as the test is sensitive to the small number of items within the scale [24], as well as the imputation of data. Only a slightly higher Cronbach’s α of 0.90 could be reached if items 2 or 3 were deleted, suggesting a high degree of item-interrelatedness.

It is a limitation that data had to be imputed to complete the dataset. In future research, a study sample where the respondents are able to answer all the items, including the ones dealing with contact between patient and health care professional (items 6–10), should be considered. Also, the generalizability of the results may be reduced by the fact that all the respondents were male, prostate cancer patients and limited to those between the ages of 54–73. Furthermore, it has to be taken into consideration that even though the sample size is accurate to test the EFA, a larger sample size is preferable.

Psychometric testing is often left out when a questionnaire is being used, and the fact that some initial testing has been performed is an obvious strength. Furthermore, the questionnaire may be a valuable tool to assess whether or not a given PROM-questionnaire should be implemented in the clinic or to assist clinicians in choosing which questionnaire to use in a given context. There is a need to “capture patient’s experience of treatment and care as a major indicator of health service quality and treatment effectiveness” [25]. Using the Patient Feedback Form may be a possibility. Moreover, future studies including PROMs can be improved by using the present PREM-instrument, which is now available in Danish, allowing researchers and clinicians to measure patient satisfaction parallel to PROMs [4] and compare results nationally and internationally.

## Conclusion

The translated, culturally adapted, and validated Danish version of the Patient Feedback Form seems to be suitable for measuring satisfaction with PROMs in this prostate cancer population. To further verify the validity of the instrument, the next step should be psychometric testing such as hypotheses testing, responsiveness, and test-retest on a larger and more diverse sample size.

## Abbreviations

EFA: Exploratory factor analysis
EM: Expectation-maximization
KMO: Kaiser-Meyer-Olkin
MCAR: Missing Completely at Random
PREM: Patient-reported Experience Measures
PROM: Patient-reported Outcome Measures
QoL: Quality of life
